# Microbubble-Protected Oncolytic Virotherapy Targeted by Sonoporation Induces Tumor Necrosis and T-Lymphocyte Infiltration in Humanized Mice Bearing Triple-Negative Breast Cancer

**DOI:** 10.3390/ijms252413697

**Published:** 2024-12-21

**Authors:** Juliana Sitta, Flavia De Carlo, Imani Kirven, John H. Tackett, Patrice Penfornis, George Clement Dobbins, Mallory Barbier, Luis Del Valle, Clayton T. Larsen, Ernest G. Schutt, Rhodemann Li, Candace M. Howard, Pier Paolo Claudio

**Affiliations:** 1 Department of Radiology, University of Mississippi Medical Center, Jackson, MS 39216, USA; jsitta@umc.edu (J.S.); cmhoward@umc.edu (C.M.H.); 2 Department of Biomedical Sciences, Imaging Track, University of Mississippi Medical Center, Jackson, MS 39216, USA; 3 Department of Pharmacology & Toxicology, Cancer Center & Research Institute, University of Mississippi Medical Center, Jackson, MS 39216, USA; flavia.de-carlo@chuv.ch (F.D.C.); ikirven@umc.edu (I.K.); jtackett1@umc.edu (J.H.T.); ppenfornis@umc.edu (P.P.); 4 Department of Neurosurgery and Bioinformatics, University of Alabama Birmingham, Birmingham, AL 35205, USA; gclemd@uab.edu; 5 Department of Pathology, Louisiana Cancer Research Center, Louisiana State University Health, New Orleans, LA 70112, USA; mbarbi@lsuhsc.edu (M.B.); ldelva@lsuhsc.edu (L.D.V.); 6 Vesselon, Inc., Norwalk, CT 06851, USA; clay.larsen@vesselon.com (C.T.L.); eschutt@san.rr.com (E.G.S.); rhodemann.li@vesselon.com (R.L.)

**Keywords:** oncolytic virus, gene therapy, microbubbles, microspheres, ultrasound contrast agent, ultrasound, cavitation, immunotherapy

## Abstract

Oncolytic virotherapy has shown great promise in mediating targeted tumor destruction through tumor-selective replication and induction of anti-tumor immunity; however, obstacles remain for virus candidates to reach the clinic. These include avoiding neutralizing antibodies, preventing stimulation of the adaptive immune response during intravenous administration, and inducing sufficient apoptosis and immune activation so that the body’s defense can work to eradicate systemic disease. We have developed a co-formulation of oncolytic viruses (OVs) with Imagent^®^ lipid-encapsulated, perfluorocarbon microbubbles (MBs) to protect the OVs from the innate and adaptive immune system. Once inside the MB, the viral particles become acoustically active such that external ultrasound can target the delivery of the virus locally within the tumor. Humanized NSG female mice (Hu-CD34^+^ NSG-SGM3) engrafted in their flanks with MDA-MB-231-Luc triple-negative breast cancer (TNBC) cells were transduced with MB/OVs, with or without adjuvant Pembrolizumab treatment, and tumor sizes and tumor necrosis were assessed. The presence of CD8^+^ (cytotoxic T-cells), CD4^+^ (helper T-cells), and CD25^+^ (Tregs) tumor-infiltrating lymphocytes (TILs) was quantified in the tumor samples by immunohistochemistry. In an in vivo model of humanized mice engrafted with a human immune system, we observed significantly greater tumor necrosis and smaller tumor mass in human TNBC xenografts systemically treated with MB/OV complexes in the presence or absence of pembrolizumab adjuvant treatment, compared to controls. Additionally, we observed a low ratio of CD4^+^/CD8^+^ TILs and a high ratio of CD8^+^/CD25^+^ TILs in the MDA-MB-231 xenografts treated with MB/OVs complexes with or without pembrolizumab adjuvant treatment, compared to controls. Our study demonstrated the feasibility of using MBs to target OVs to TNBC through diagnostic ultrasound, which decreased tumor mass by increasing tumor necrosis and stimulated a local and systemic antitumoral immune response by increasing intratumoral CD8^+^ T-cytotoxic lymphocyte infiltration and decreasing CD25^+^ Treg cells.

## 1. Introduction

Several advances in the development of therapeutic interventions, such as chemotherapy, radiation therapy, and immunotherapy, have led to improvements in the prognosis of cancer patients. However, these treatments have shown significant limitations due to serious adverse events, the development of therapy resistance, and the unreliable efficacy of immunotherapy in immune-depressed subjects [1]. For these reasons, novel therapeutic approaches have been developed to avoid unnecessary adverse effects on normal tissue cells while increasing cancer cell specificity, including the development of oncolytic virotherapy.

Oncolytic viruses (OVs), which constitute a group of viruses found in nature as well as genetically modified viruses that can replicate in cancer cells while sparing normal cells, are among new therapeutic approaches recently developed. The OVs have an elevated dependence on tumor signaling pathways, which makes their replication selective, allowing for targeted infection and/or intracellular proliferation within tumor cells [1]. The main antitumoral mechanisms elicited by OVs during their life cycle are direct and indirect. The direct antitumor effect on the infected cancer cells is caused by the burst of the cancer cells with the release of newly assembled OVs that amplify and expand the initial antitumoral activity. The indirect antitumor effect is due to the release of peptide fragments from the lysed tumor cells that stimulate the innate and adaptive immune response, ultimately resulting in additional tumor clearance.

The only oncolytic virus approved in the U.S. by the Food and Drug Administration (FDA), albeit back in 2015, is talimogene laherparepvec (T-VEC), a modified attenuated herpes simplex virus, for treating advanced inoperable melanoma [1]. As a superficial disease, melanoma is easily accessible to the intratumoral (IT) injections T-VEC requires. However, IT injections are challenging for other solid tumors for widespread clinical adoption. Variations in injection technique and injection needle design, as well as tumor microenvironmental factors such as tumor interstitial pressure, can result in dramatic alterations in intratumoral viral distribution. From a purely practical perspective, previous studies conducted have demonstrated that when oncolytic viruses are injected directly into a tumor, the infection of the oncolytic virus is limited to a portion of the tumor even if the injected volume is sufficient to cover the entire tumor [2,3]. Additionally, therapies that are best suited for weekly administration such as virotherapy would increase the risk of bleeding and infection. At the same time, deeper tumors require sedation with associated risks and inconvenience for the patient. These logistical challenges have limited the therapeutic adoption of OVs [4,5].

The field of microbubble-mediated, ultrasound-targeted delivery of therapeutics holds great promise to non-invasively guide viruses and drugs to any tumor within the body [6,7], including those that are not superficial. The biophysical effect of sonoporation at the tumor can provide multi-fold increases in tumor biodistribution far beyond what the unaided therapeutic index of a drug can offer [8]. When this targeted microbubble (MB) drug delivery platform concept is applied to viruses, it can avert the need for a multiplicity of tumor-specific tropism candidates as the biophysical interaction of the MBs solves both targeting and transduction efficiency challenges. Viral candidates can then be chosen based on their cytotoxicity and the gene payload delivered for selective protein expression. Under certain circumstances, MBs can include the added benefit of particle encapsulation, which protects the virus from both adaptive and innate immune responses in the systemic circulation when traveling to the tumor [9]. Additionally, it has also been demonstrated that this MB-mediated technique is superior to IT techniques in terms of heterogeneous dose distribution across the tumor [10].

The systemic site-specific delivery system we developed uses ultrasound (US) contrast agents as the delivery vehicles, here referred to as MBs. The viruses are loaded inside lipid shells of acoustically active, lyophilized, perfluorocarbon-filled MBs (Imagent^®^), which, once destroyed at the tumor site by diagnostic-grade US, release the OVs to the targeted tissue. The MBs range between 1.0 and 6.0 µm in diameter, and, after injection into the bloodstream, they can re-circulate through the vascular system numerous times [3,11,12]. Their small dimension prevents entrapment within the pulmonary capillary bed (~5 to 8 µm) [3]. We have successfully utilized this MB gene transfer system to selectively transfer both expression markers and/or therapeutic genes into tumors in immune-deficient mice [3,13,14,15]. We also demonstrated previously that the MBs protect a viral payload from detection and rapid degradation by the hosts’ immune system, allowing for an intravenous (IV) inoculation rather than an IT injection [3,16]. Additionally, we did not observe activation of innate (TNF-α and IL-6 cytokines) or adaptive immune response (neutralizing antibodies, INF-γ + CD8^+^ T cells) in healthy or tumor-bearing immunocompetent C57BL/6 mice injected with MB/virus complexes following ultrasound-targeted MB destruction (UTMD) [9].

In our study, we examined the therapeutic effects of a synthesis of technologies that work in concert to optimize a drug delivery system using a third-generation oncolytic virus to target an in vivo TNBC xenograft model in humanized mice (Hu-CD34^+^ NSG-SGM3) with or without checkpoint inhibitor combination therapy to understand the role of ultrasound-targeted destruction of MB/OV complexes on the immune system and its therapeutic impact on the recruitment of CD4^+^ and CD8^+^ TILs causing tumor clearance.

## 2. Results

### 2.1. Microbubble-Assisted Delivery of Oncolytic Adenoviruses Targets MDA-231-MB Human Triple Negative Breast Cancer In Vivo

For our studies, we used Hu-CD34^+^ NSG-SGM3, which are NSG-SGM3 mice (NOD.Cg-Prkdcscid Il2rgtm1Wjl Tg(CMV-IL3, CSF2, KITLG)1Eav/MloySzJ, Stock No. 013062) engrafted with human cord blood-derived CD34^+^ hematopoietic stem cells. Forty-eight Hu-CD34^+^ NSG-SGM3 mice were injected with MDA-MB-231-luc human TNBC cells in their flanks (right and left flanks, 5 × 10^6^ cells/mouse/flank in Saline: Matrigel Growth Factor Reduced 1:1). When tumors reached 150–200 mm^3^ by caliper measurement, mice were randomized by body weight and assigned to one of the following six groups: (1) CTRL MBs (IV); (2) OVs (IT); (3) OVs (IV); (4) Pembrolizumab (IP); (5) OVs/MBs (IV); (6) OVs/MBs (IV) + Pembrolizumab (IP) (Table 1). Once assigned to the groups, mice ears were punctured for identification.

**Figure 1:**
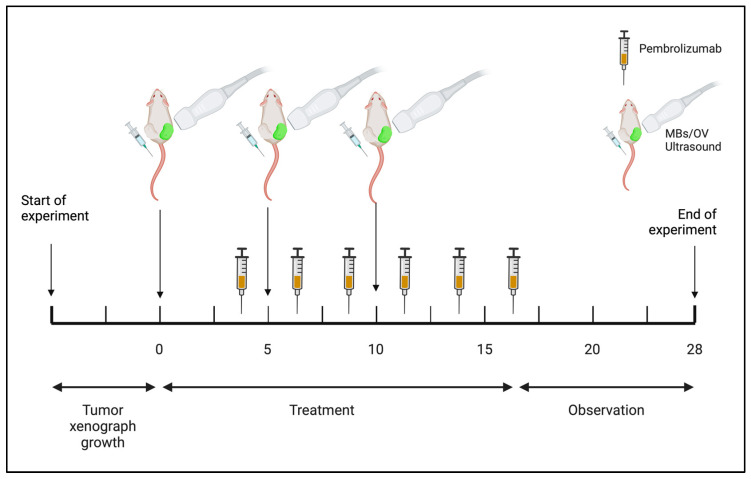
Study treatment schedule. Treatments started when tumors were 150 mm^3^. Created with Biorender.com.

Anesthetized mice were treated according to the randomization group of assignment (Table 1) and with the schedule as depicted in Figure 1.

A total of 2.8 × 10^10^ PFU of the oncolytic Adenoviruses Ad5/3-C-RGD-D24 were used to reconstitute the vials of Imagent^®^ to obtain an average suspension of 1.37 × 10^10^ MBs. The viruses adsorbed on the MB surface were removed by treating the MBs with ten volumes of human serum complement at 60 mg/mL (Creative Biolabs, Shirley, NY, USA, CTS-006) and then washing the MBs into sterile saline before injection [13]. The final MB preparation (300 µL) was counted using a hemocytometer (Table 2) and diluted with 2 mL of saline to a concentration of 5 × 10^7^ PFU/100 µL. One aliquot of each of the preparations was used to titrate the OVs post-encapsulation in the MBs (Table 3).

Mice in Groups 1, 2, 3, 5 and 6 received a total of three injections, one dose every five days (Q5Dx3) of saline control (IV), OVs (IT), OVs (IV), or OV/MBs. The mice in groups 2 and 3 received 5 × 10^7^ PFU/mouse/50 µL of OVs by IT or IV as a control. The mice in groups 5 and 6 received 9.8 × 10^4^ PFU/100 µL/mouse of MBs/OVs for the first two injections and 1.09 × 10^8^ PFU/100 µL/mouse of MBs/OVs for the third injection. The mice in groups 4 and 6 received six intraperitoneal (IP) injections, one dose every five days (Q5Dx6) of Pembrolizumab (Selleckchem, Houston, TX, USA, anti PD-1, A2005). The first Pembrolizumab injection was administered at 10 mg/kg in saline and the following injections were performed using a dose of 5 mg/kg in saline, as previously [17].

### 2.2. Microbubble-Assisted Delivery of Oncolytic Adenoviruses Increases the Necrosis but Not the Size of MDA-231-MB Human Triple Negative Breast Cancer In Vivo

**Figure 2:**
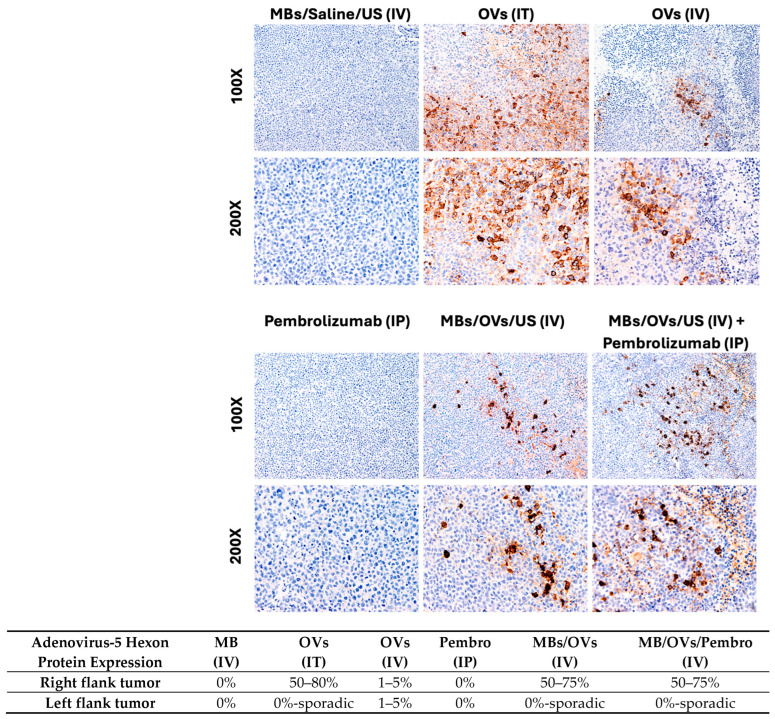
Detection of Adenovirus-5 immunohistochemistry. Adenovirus-5 Hexon protein expression was negative in the controls (MBs and Pembrolizumab groups). Adenovirus-5 Hexon expression significantly increased in the OV group, with a 50–75% rate in infected cells in the IV groups. The magnification of the upper panels is 100×, and the lower panels is 200×.

At the experimental endpoint (4 weeks from the first treatment), mice were sacrificed, and tumors, lungs, heart, liver, and kidneys were collected, and fixed in 10% neutral buffered formalin for 72 h. No liver, heart, kidney, or lung inflammatory damage was found by H&E staining. Immunohistochemistry (IHC) for the Hexon protein of adenovirus-5 was performed on tissue sections to detect the presence of OVs delivered to the TNBC xenografts. Figure 2 shows the prominent adenovirus-5 Hexon protein expression in TNBC xenografts directly injected IT with the OVs and in xenografts treated with MB/OV complexes in the presence of US.

**Figure 3:**
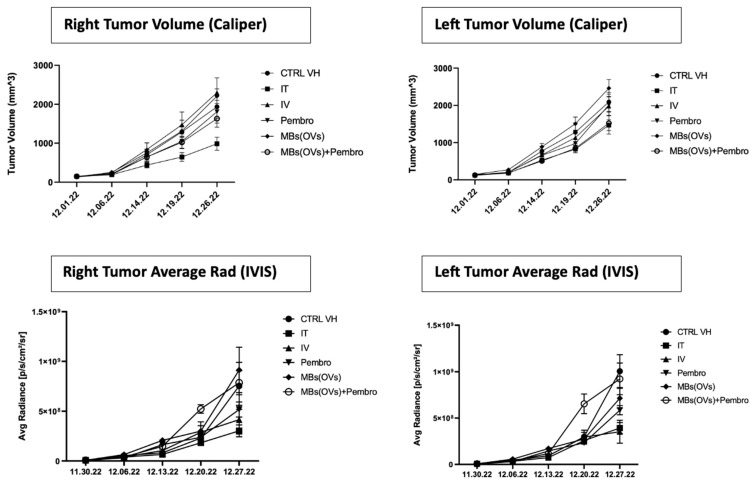
Tumor measurements by caliper and by IVIS.

Tumors were measured once a week using a digital caliper, and the volume was calculated by the modified ellipsoidal formula: V = ½ (Length × Width^2^). Tumor volumes were also estimated by IVIS imaging by measuring ROI Avg Radiance [p/s/cm^2^/sr]. We did not observe a statistical difference in the tumor volumes and in the weight of the treated vs. control animals when measured by caliper and/or ROI average radiance (Figure 3). Mice were weighed once a week. We did not detect significant differences in the weight of mice in the various groups. The greatest difference we found was between control mice and mice treated with MB/OV complexes in the presence of adjuvant Pembrolizumab.

**Figure 4:**
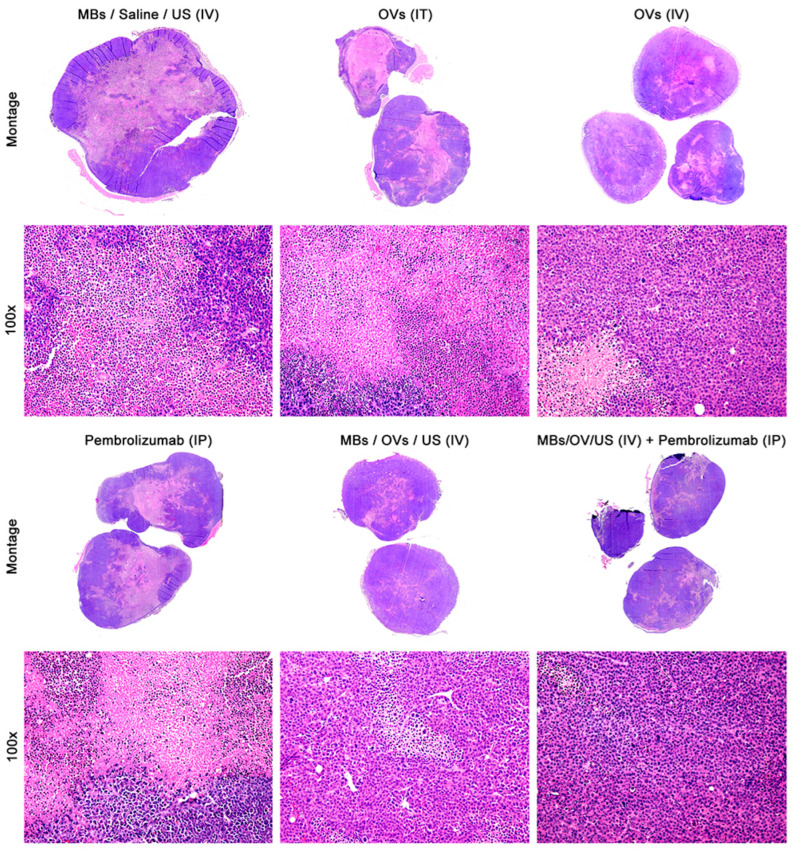
Hematoxylin and eosin staining of tumor xenografts (right flank). A montage of the treated tumor is shown on the upper panels, where the areas of necrosis are evident; lower panels show a low magnification view of the tumors in which the different sizes of the necrotic areas can be observed (original magnification 100×).

Hematoxylin and eosin (H&E) staining was performed on tissue sections to detect necrosis (Figure 4). A pathologist evaluated H&E-stained tumors, measuring their size and determining the amount of necrosis.

The most extensive necrosis was observed in tumors on the right flank treated with MB/OV complexes + US and adjuvant Pembrolizumab, followed by xenografts treated with MB/OV complexes + US (Figure 4 and Table 4).

Interestingly, an abscopal effect was observed on tumor necrosis in the contralateral untreated tumors (left flank tumors), in mice injected with MB/OV complexes + US with or without adjuvant Pembrolizumab treatment (Figure 4 and Table 4).

A multivariate ANOVA comparison with post hoc Tukey of all groups showed that a statistically significant difference was found comparing the tumor size/necrosis ratio of right flank xenografts treated with intratumoral injections of naked OVs vs. the tumor size/necrosis ratio of right flank xenografts treated with MB/OV complexes + Pembrolizumab (* *p* < 0.05; CI_95_ = 3.94–7.91).

A statistically significant difference was also found by comparing by one-tailed *t*-test the tumor size/necrosis ratio of tumors on the right flank treated with MB/OV complexes + US + adjuvant Pembrolizumab to control MB-treated xenografts (* *p* < 0.05), and at a lower extent by comparing the tumor size/necrosis ratio of xenografts treated with MB/OV complexes + US to control MB treatments (*p* = 0.05) (Table 5). Statistically significant differences were also found comparing the tumor size/necrosis ratio of tumors on the right flank treated with IT injections using naked OVs to intravenous injections of naked OVs (* *p* < 0.05), or intravenous injections of MB/OV complexes + US (* *p* < 0.05), or intravenous injections of MB/OV complexes + US and adjuvant Pembrolizumab (* *p* < 0.05) (Table 5). Other statistically significant differences were found comparing the tumor size/necrosis ratio of TNBC xenografts treated with intravenous injections of naked OVs to treatments with MB/OV complexes + US + adjuvant Pembrolizumab (* *p* < 0.05), and of treatments with pembrolizumab alone compared to intravenous injections of MB/OV complexes + US + adjuvant Pembrolizumab (* *p* < 0.05) (Table 5).

The abscopal effect we observed on tumor necrosis in the contralateral untreated tumors (left flank tumors) of mice injected with MB/OV complexes + US with or without adjuvant Pembrolizumab treatment (Table 4) was further analyzed by comparing the tumor size/necrosis ratio of the various groups. One-tailed T-test comparisons of the tumor size/necrosis ratio of untreated xenografts on the left flank to intravenous injections of MB/OV complexes + US showed a statistically significant *p*-value of less than 0.05 and at a lower extent when compared to mice treated with MB/OV complexes + US and Pembrolizumab (* *p* = 0.05) (Table 6). Additionally, statistically significant differences were found by comparing the tumor size/necrosis ratio of non-sonoporated tumors on the left flank injected with MB/OV complexes + US with or without adjuvant Pembrolizumab treatment to mice treated with naked OVs injected intravenously (* *p* < 0.05) (Table 6).

**Figure 5:**
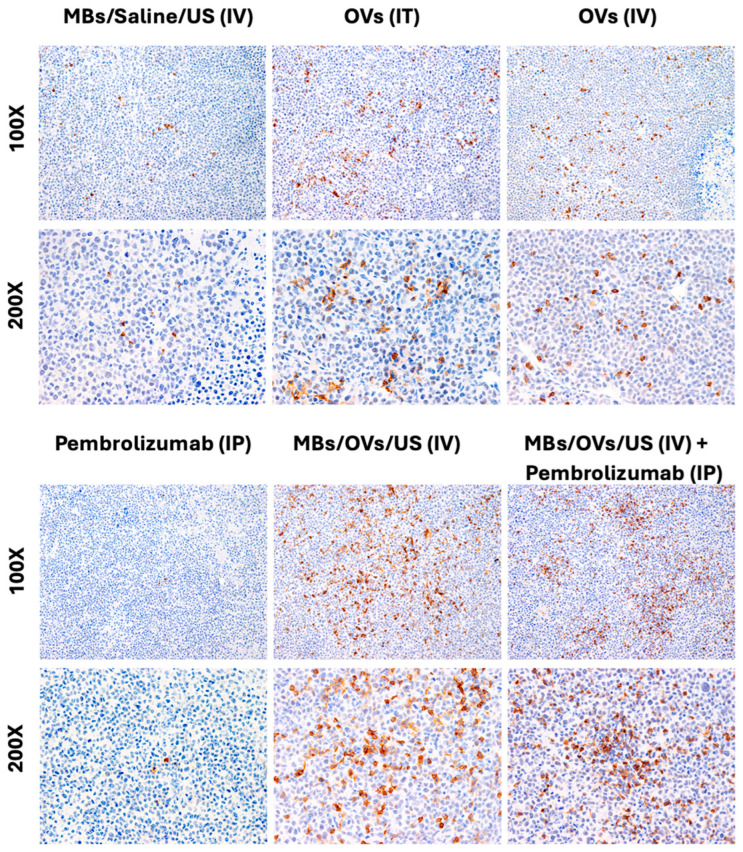
Detection of tumor-infiltrating CD4^+^ cells by immunohistochemistry. CD4-positive immune cells are few in the control (saline) group and sporadic in the Pembrolizumab-treated group, but their numbers significantly increase in the OV groups and dramatically increase in the MB groups. The upper panel magnification is 100×, and the lower panel magnification is 200×.

**Figure 6:**
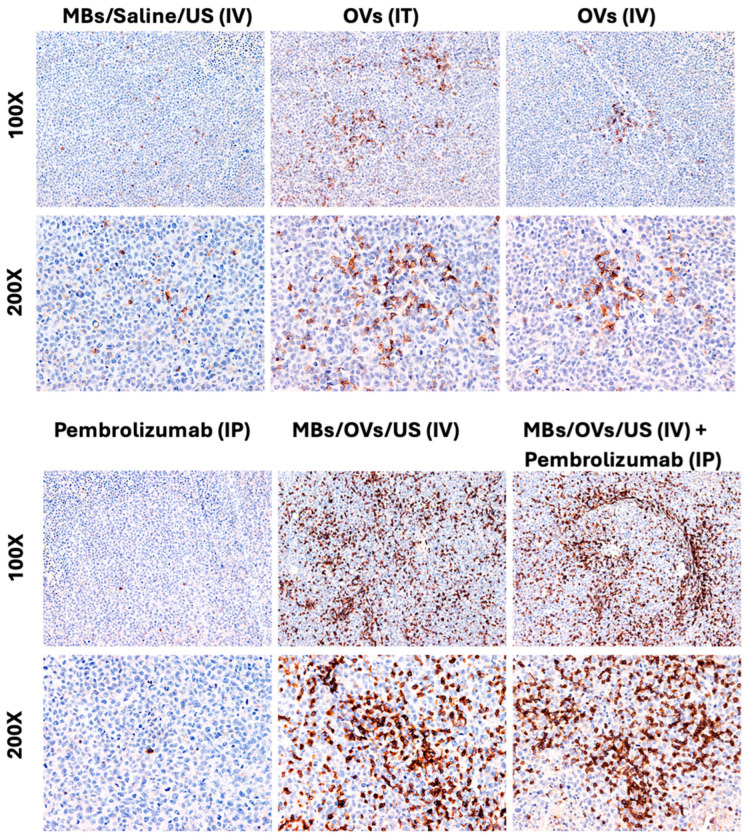
Immunohistochemical detection of CD8^+^ T-cells in tumors. CD8^+^ T cell tumor infiltrates (TILs) are few in the control (saline) group, and sporadic in the Pembrolizumab treated group; however, their expression significantly increases in both OV groups, and their number is dramatically increased in both MB groups. The magnification of the upper panels is 100×, and the magnification of the lower panels is 200×.

Immunohistochemical analysis with antibodies against human CD8^+^ and CD4^+^ was performed on tissue sections to characterize the T lymphocytes infiltrating the tumors (Figure 5 and Figure 6).

We observed an increase in CD4^+^ tumor-infiltrating T-cells (TILs) in the MDA-MB-231 xenografts treated with MB/OVs complexes with or without pembrolizumab adjuvant treatment compared to mice injected with unprotected OVs (IT or IV) or IV with control MBs (Figure 5). A multivariate ANOVA comparison analysis with Tukey multiple comparisons post-test determined that tumors on the right flank treated with MB/OV complexes with or without pembrolizumab adjuvant treatment compared to control mice injected with MB alone had a highly statistically significant increase in CD4^+^ recruitment (** *p* < 0.01). Also, tumors on the right flank treated with MB/OV complexes with or without pembrolizumab adjuvant treatment compared to intravenous injections of naked OVs had a highly statistically significant increase in CD4^+^ recruitment (* *p* < 0.05 and ** *p* < 0.01, respectively) (Table 7). No statistical differences in the recruitment of CD4^+^ TILs in untreated tumors (left flanks) were found instead among the various groups of mice.

A greater increase in CD8^+^ TILs was observed in the MDA-MB-231 xenografts treated with MB/OVs complexes with or without pembrolizumab adjuvant treatment compared to mice injected with unprotected OVs (IT or IV) or IV with MB control (Figure 6).

A multivariate ANOVA comparison analysis with Tukey multiple comparisons post-test determined that tumors on the right flank treated with MB/OV complexes with or without pembrolizumab adjuvant treatment compared to any of the control groups of mice had a highly statistically significant increase in CD8^+^ recruitment (** *p* < 0.01). Also, tumors on the right flank that were injected intratumorally with naked OVs had a statistically significant increase in CD8^+^ TILs compared to Pembrolizumab treatments or to control MB injections (** *p* < 0.01) (Table 8).

A multivariate ANOVA comparison analysis with Tukey multiple comparisons post-test determined that untreated tumors on the left flank of mice treated on the right flank tumors with MB/OV complexes with or without pembrolizumab adjuvant treatment compared to pembrolizumab injections or to IT injections of naked OVs had a highly statistically significant increase in CD8^+^ recruitment (** *p* < 0.01 and * <0.05, respectively) (Table 9).

It is well known that patients with TNBC with greater tumor infiltrates of CD8^+^ than CD4^+^ have better outcomes following therapy. We compared the ratio of CD4^+^ and CD8^+^ cell types (helper and cytotoxic T lymphocytes, respectively) in the TILs in the various groups (Figure 5 and Figure 6, and Table 10). We observed a lower ratio of CD4^+^ to CD8^+^ TILs in the TNBC xenografts of mice injected with MB/virus complexes following sonoporation and in the presence or absence of a Pembrolizumab treatment, compared to the controls (Table 10). The difference between the CD4^+^/CD8^+^ ratio of tumors on the right flank treated with MB/OV complexes + US in the presence or absence of adjuvant Pembrolizumab compared to control xenografts treated with intratumoral injections of the OVs was found to be statistically significant using a one-tailed *t*-test (* p < 0.05). No statistically significant difference was found instead between the CD4^+^/CD8^+^ ratio of untreated tumors on the left flank of mice treated with MB/OVs complexes + US in the presence or absence of adjuvant Pembrolizumab compared to control xenografts treated with intratumoral injections of the OVs.

**Figure 7:**
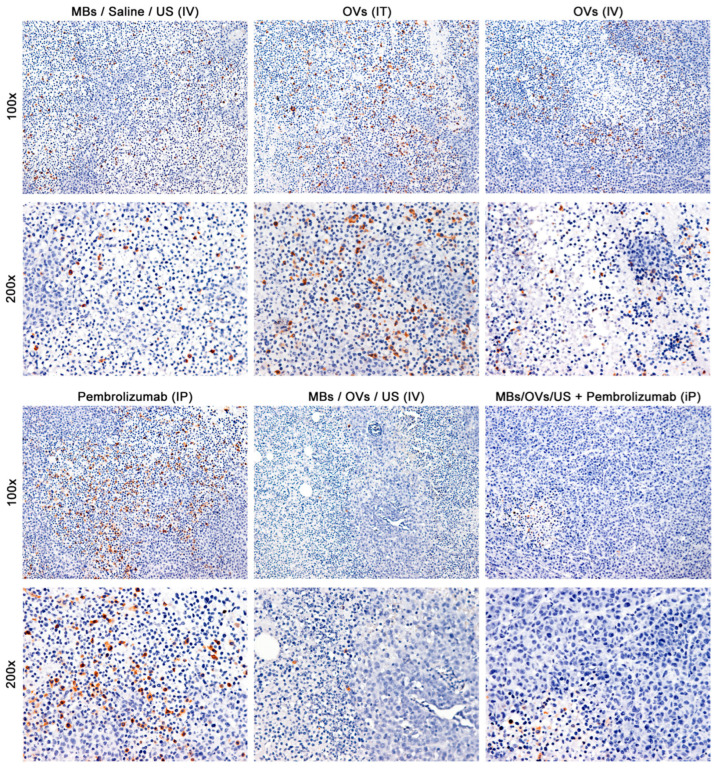
Immunohistochemistry for IL-R2 alpha (CD25) in tumors. CD25^+^ cells are regularly distributed in the control MB group and are abundant in the Pembrolizumab group, mainly located within or surrounding areas of necrosis; however, their number plummeted in both MB groups, coinciding with these tumors having significantly smaller areas of necrosis. The magnification of the upper panels is 100×, and the magnification of the lower panels is 200×.

It has also been shown that patients with TNBC with fewer CD25^+^ (Tregs) tumor infiltrates than CD8^+^ (Cytotoxic) T-cells have better outcomes following therapy. We compared the ratio of CD8^+^ to CD25^+^ cells in the TILs in the different treated groups (Figure 6 and Figure 7, Table 10). We observed a high ratio of CD8^+^ to CD25^+^ TILs in the TNBC xenografts of mice injected with MB/OV complexes in the presence or absence of pembrolizumab adjuvant treatment following sonoporation, compared to mice injected IT or IV with unprotected OVs or with MB control (Table 10). In addition, unlike CD4 and CD8 cells, which are infiltrating tumor cells, IL2Ra-positive cells are mainly located within and around areas of necrosis, which is expected of regulatory cells.

A multivariate ANOVA comparison analysis with Tukey post-test of the CD8^+^/CD25^+^ ratio of TILs in tumors on the right flank treated with MB/OV complexes in the presence of pembrolizumab adjuvant treatment compared to any groups of mice was highly statistically significant (** *p* < 0.01) (Table 11).

Also, tumors on the right flank treated with MB/OV complexes in the absence of pembrolizumab adjuvant treatment were found to be highly statistically significant (** *p* < 0.01) when compared to MB or IT control and to pembrolizumab treatments. No statistical differences in the ratio of CD8^+^/CD25^+^ TILs in untreated tumors (left flanks) were found instead among the various groups of mice.

## 3. Discussion

The recent FDA approval of Imlygic (Talimogene laherparepvec, T-VEC) 40 and the development of other oncolytic viruses in clinical trials highlight the promise of this new class of anti-cancer agents. Oncolytic viruses are mainly injected intratumorally to provoke innate immune responses in the tumor site, converting the immunosuppressive TME to an immunogenic one. If administered systemically, oncolytic viruses (OVs) are subject to neutralizing antibodies [18], which limits their repeated administration, causing highly variable clinical responses [6,7,19]. The innate immune system represents the body’s first line of immune response to OV treatment, which determines to what extent tumors become affected by these agents [20], to what degree an inflammatory response within the tumor microenvironment (TME) develops [21], and ultimately whether long-term tumor-specific adaptive immunity is stimulated [22,23]. The presence of an immunosuppressive TME and immune escape are the main reasons that can make immunotherapy ineffective.

Oncolytic viruses have great potential to work alone or in combination with immunotherapies to better control cancer, particularly those that are unresponsive to immune checkpoint therapy [24]. Turning immunologically “cold” tumors “hot” by improving T-cell infiltration is becoming increasingly investigated, especially because new evidence supports the idea that the immunogenicity of OVs may be more important than any direct oncolytic activity [25]. Cancer immune surveillance is a concept that originated in the nineteenth century [26] and is a significant process by which the immune system can identify and eliminate cancer cells [27]. Compared with previous standards of care (including chemotherapy, radiotherapy, and surgery), cancer immunotherapy has significantly improved patients’ survival and quality of life, although immunotherapy medications used in cancer can cause devastating adverse events such as life-threatening reactions.

It is generally believed that intratumoral immunotherapy and oncolytic virotherapy could be more effective and reduce systemic toxicities by increasing agent bioavailability inside tumors [5,28,29,30]. However, intratumoral injections of immunotherapy or OVs are not always feasible. Recent experience demonstrates that intratumoral therapies have had limited success in late-phase trials for advanced cancer, resulting in few formal regulatory approvals [31]. Alternative non-invasive technologies that can outperform intratumoral injections in terms of biodistribution, greater immune stimulation, and similar if not lower adverse effects are greatly needed. The ideal immunotherapy drug and delivery system should have several characteristics, including (1) being non-invasive, (2) targeting solid tumors anywhere in the body, (3) stimulating an effective immune response in the tumor, (4) increasing apoptotic tumor cell death, (5) causing minimal systemic adverse events, and (6) treating metastatic disease.

Sonoporation is a targeted delivery technique that uses ultrasound to enhance the permeability of cell membranes and promote the localized delivery of therapeutic agents, such as drugs, genes, or oncolytic viruses. This process is facilitated by US contrast agents (MBs), which are small, gas-filled spheres encapsulated by lipid or protein shells. When exposed to ultrasound, MBs oscillate due to the alternating pressure of the sound waves, resulting in a series of mechanical effects that increase the cellular uptake of therapeutic agents. The two main types of cavitation associated with sonoporation are stable cavitation and inertial cavitation, each of which contributes uniquely to the process of targeted delivery [32].

In stable cavitation, which occurs at a lower mechanical index, MBs undergo controlled oscillations causing repeated expansion and contraction without collapsing. This oscillatory behavior generates localized microstreaming, where small, high-velocity fluid currents form around the oscillating MBs. The mechanical forces produced by microstreaming can disrupt the cell membrane temporarily, creating small pores that allow therapeutic agents to enter the cell. Importantly, stable cavitation facilitates drug delivery while minimizing cellular damage, making it a gentle yet effective approach for enhancing membrane permeability [33]. This process is particularly valuable in tumors, where increasing the local cellular uptake of therapeutic agents can significantly enhance treatment efficacy.

In contrast, inertial cavitation, which occurs at a higher mechanical index, involves the rapid collapse of MBs. During inertial cavitation, MBs expand rapidly and then implode, releasing substantial energy in the form of shock waves and high shear forces. This biophysical mechanical action creates larger, transient pores in cell membranes and can even disrupt the extracellular matrix surrounding tumor cells, improving the diffusion and distribution of therapeutic agents within solid tumors [33].

The effectiveness of sonoporation is further improved by the enhanced permeability and retention (EPR) effect. Due to rapid and disorganized growth, tumor blood vessels are often abnormally leaky, which allows MBs and their therapeutic payloads to accumulate more readily within tumor tissue. Sonoporation can increase the permeability of these vessels, further enhancing the local retention of therapeutic agents in the tumor [3].

Our study builds on this technique by combining oncolytic viruses (OVs) with MBs to achieve targeted delivery in a TNBC model. As shown in Figure 2, our results confirm that ultrasound-triggered MB/OV complexes enabled effective targeted viral release and accumulation within the treated tumor, evidenced by a significant increase in viral presence and tumor necrosis only in treated right flank tumors compared to the untreated left flank tumors and control groups. In this study, we developed a humanized tumor-bearing mouse model of TNBC to investigate Adenovirus-mediated oncolytic virotherapy and checkpoint inhibition in the context of the human immune system by using ultrasound contrast agents in the form of microbubbles to shield the OVs from the host immune system and achieve site-specific targeted therapy. CD4^+^ and CD8^+^ T-lymphocyte tumor infiltrates, which are the most powerful effectors in anticancer immune response and constitute the backbone of cancer immunotherapy, were investigated [34]. Additionally, we investigated the presence of CD25^+^ regulatory T cells (Tregs) that protect cancer cells from the cytotoxic activity of CD8^+^ T-cells [35,36,37,38,39].

Our data demonstrated that sonoporation of MB/OV complexes elicited significantly more tumor necrosis and a robust increase in CD8^+^ T-lymphocyte tumor infiltration with a low ratio of CD4^+^/CD8^+^ TILs, which is independent of PD-1 checkpoint inhibitor treatments, indicating a favorable prognostic outcome of oncolytic virotherapy. These findings agree with several human studies of TNBC that showed a low ratio of CD4^+^/CD8^+^ as a positive prognostic indicator of increased immunity against the tumor [38,40,41]. This strong therapeutic effect also appeared to benefit the opposite flank tumors that were not treated with ultrasound, indicating a systemic abscopal effect not elicited by IT and IV dosing of unprotected OVs. The use of a biophysical aid, such as ultrasound from a diagnostic-grade US machine that creates shockwaves that remodel the tumor microenvironment (TME) and enhance the permeability and retention (EPR) of the OVs in the targeted tissue [42], enhances drug biodistribution of the MB payload to tumor cells and has great promise.

In general, TNBC stands out among other breast cancers (BCs) due to its occurrence in younger women, higher grade, aggressive clinical course, frequent lymph node and distant metastases, and significant mortality [43]. TNBC is also considered to have the highest immunogenic potential and abundance of TILs of all subtypes of BC [44]. The potential prognostic value of TILs in TNBC has been extensively investigated [45], and several studies addressing the issue of tumor immune cell infiltration have consistently demonstrated that a high lymphocytic infiltration predicts a better prognosis [46].

Research has consistently shown a significant relationship between CD4^+^ T cell infiltration and TNBC, with CD4^+^ T cells playing crucial roles in modulating tumor progression and shaping the immune response. CD4^+^ T cells primarily function as helper cells, assisting in orchestrating the immune response through secretion of interleukin-2 and interferon-gamma, which support the proliferation and enhance the cytotoxicity of CD8^+^ cells [47]. Additionally, Th1-polarized CD4^+^ cells release cytokines such as interferon-gamma and tumor necrosis factor-alpha, which improve antigen presentation by dendritic cells and enhance tumor cell recognition. This Th1 polarization is particularly beneficial in TNBC, where increased immunogenicity can make tumors more responsive to immunotherapy. The persistence of CD8^+^ T cells is also linked to CD4^+^ T cell-derived signaling, which helps maintain cytotoxic activity over extended periods, crucial for sustained tumor suppression [38].

Studies have observed that treatments increasing CD4^+^ T cell infiltration in TNBC can amplify the anti-tumor immune response, particularly when CD4^+^ cells foster CD8^+^ T cell proliferation, differentiation, and retention within the tumor microenvironment [48]. These findings align with our study, which observed better outcomes in mice treated with MB/OV complexes, with or without pembrolizumab adjuvant treatment, compared to mice injected with unprotected OVs (intratumoral or intravenous) or control MBs. This positive response was associated with an increase in CD4^+^ TILs.

Intratumoral CD8^+^ cytotoxic T cells play an important role in tumor-killing activity by interacting with tumor antigens and causing direct or indirect cell lysis mediated by cytokines such as interferon-γ, tumor necrosis factor α (TNFα), and granulocyte-macrophage colony-stimulating factor (GM-CSF) [49]. A higher representation level of CD8^+^ TILs has been significantly associated with breast cancer-specific survival in a study of 1334 unselected breast tumors from patients with long-term follow-up [50]. CD4^+^ and CD8^+^ T-cell immune infiltrations are associated with higher odds for pathological complete response (CR) in a study of 195 patients with TNBC [51]. In several studies, a high number of CD4^+^ and CD8^+^ TILs were also associated with a lower risk of mortality and recurrence in TNBC [52,53,54,55]. Additionally, intratumoral CD8^+^ lymphocytes were found to be independently associated with breast cancer-specific survival in TNBC [56].

Prior research has highlighted the importance of a low CD4^+^/CD8^+^ TIL ratio as a favorable prognostic indicator in triple-negative breast cancer (TNBC). A lower CD4^+^/CD8^+^ ratio minimizes the presence of Tregs, favoring an immune environment conducive to direct tumor cell killing [38,41]. Tregs, characterized by CD4^+^/CD25^+^/FoxP3^+^ expression, can inhibit CD8^+^ T cell cytotoxicity by secreting immunosuppressive cytokines such as interleukin-10 (IL-10) and transforming growth factor-beta (TGF-β), thereby facilitating tumor immune evasion. Studies have shown that a lower CD4^+^/CD8^+^ ratio corresponds to less lymph node involvement, while increased CD8^+^ T cell expression is associated with a reduction in lymph node metastasis and better overall survival in TNBC [38,57].

Our findings of a low ratio of CD4^+^/CD8^+^ in mice injected with MB/virus complexes following US coupled with increased necrosis indicate a successful outcome of the MB/OV treatment that agrees with previous human studies of triple-negative breast cancer [38,40,41]. This reduced ratio correlates with increased tumor necrosis, suggesting that MB/OV treatment promoted a shift into greater cytotoxic activity in treated tumors. Ultrasound-mediated sonoporation likely contributes to this effect by promoting localized OV release and activating pro-inflammatory signals that selectively attract CD8^+^ T cells to the tumor site.

The microenvironment of breast cancer hosts a dynamic crosstalk between diverse immune system players. While cytotoxic CD8^+^ immune cells are armed to control tumor growth and metastasis, tumors strive to recruit CD25^+^ Treg immunosuppressive cells to maintain self-tolerance, impair effective immunity, and promote tumor progression [35,36,37,38,39]. Immune escape of tumor cells is a new hallmark of cancer in general and breast cancer in particular. An immune-evasive tumor microenvironment favoring the accumulation of CD25^+^ Tregs has been previously suggested [58] in TNBC, reflecting a T cell-permissive environment, particularly in this breast cancer type [59].

Recent studies provide further insights into the role of CD25^+^ Tregs in TNBC. Fattori et al. found that elevated levels of CD25^+^ Tregs within the TNBC microenvironment significantly impair CD8^+^ T cell functionality and response to PD-1 blockade, contributing to a poor prognosis. Their study suggests that selectively targeting and depleting CD25^+^ Tregs in TNBC can shift the immune balance toward a higher CD8^+^/Treg ratio, thereby enhancing CD8^+^ T cell infiltration, activity, and therapeutic response to PD-1 inhibitors [39]. Our findings of a high ratio of CD8^+^/CD25^+^ in the presence of greater tumor necrosis in mice injected with MB/virus complexes following ultrasound also agree with the successful outcome we observed in the animals treated with MB/OV complexes.

The abscopal effect has been increasingly documented in cancer research, including in models of TNBC. This phenomenon, often triggered by immune activation, highlights the potential for localized treatments like oncolytic virotherapy or radiotherapy to generate systemic anti-tumor immunity. Mechanistically, the abscopal effect often involves the induction of immunogenic cell death and the release of damage-associated molecular patterns (DAMPs), which recruit and activate antigen-presenting cells such as DCs. This immune activation leads to the presentation of tumor antigens to CD8^+^ cytotoxic T cells, priming them for systemic anti-tumor activity. For instance, a study using an oncolytic herpes simplex virus encoding IL-12 in TNBC models observed significant tumor regression at both local and distant sites, driven by CD8^+^ T cell infiltration and the recruitment of DCs that facilitate tumor antigen presentation and systemic immune activation [60].

In our study, MB/OV complexes combined with ultrasound achieved a statistically significant reduction in average tumor size/necrosis ratio in untreated left flank tumors, in keeping with findings of abscopal effect. We also observed trends in the CD4^+^/CD8^+^ and CD8^+^/CD25^+^ ratios in untreated (left flank) tumors, particularly in the MB/OV and MB/OV + Pembrolizumab treatment groups. While these differences in immune cell ratios were not statistically significant, they suggest a potential systemic immune modulation effect induced by the localized MB/OV + ultrasound treatment on the right flank. This trend aligns with the concept of the abscopal effect observed in prior studies and further studies with larger sample sizes may be necessary to confirm the statistical significance of these trends and fully elucidate the systemic immune effects of MB/OV treatment in TNBC.

While this study provides meaningful insights into the systemic immune response and potential abscopal effects triggered by MB/OV + US treatment in TNBC, several limitations should be acknowledged. A primary limitation is the short follow-up period, which may affect the interpretation of tumor necrosis and immune cell dynamics observed in both treated and untreated tumors. Immunotherapy studies often report a “flare” phenomenon in TNBC, where an initial inflammatory response, including increases in pro-inflammatory cytokines like tumor necrosis factor-alpha, can produce early necrotic effects that mimic disease progression before long-term tumor control is established [61]. This effect may obscure early assessments of treatment efficacy. Without a longer follow-up, it is difficult to determine whether the observed necrosis reflects a durable anti-tumor response or an initial immune-related flare effect that might diminish over time. Extended monitoring in future studies would clarify the persistence and therapeutic significance of these necrotic effects [61].

Additionally, the study’s focus on CD8^+^ T cells and CD25^+^ Tregs may overlook other immunosuppressive elements present in the TNBC microenvironment, such as tumor-associated macrophages and myeloid-derived suppressor cells. These cell types are recognized for their roles in supporting immune escape and could impact the treatment outcomes observed. Broader immune profiling would help in understanding the comprehensive impact of MB/OV + US on the TME, as seen in previous studies where tumor-associated macrophages and myeloid-derived suppressor cells significantly altered the efficacy of immune-based therapies in TNBC [62]. Previous sonoporation studies have documented a beneficial reduction in MDSCs associated with MB-mediated STING agonist delivery [8].

Finally, the use of only one TNBC model limits the broader application of these findings. TNBC is a highly heterogeneous disease, and different stromal and immune cell populations within TNBC subtypes can significantly affect immune evasion and therapeutic outcomes. Wu et al. demonstrated that stromal cell diversity within TNBC plays a critical role in immune evasion, shaping the tumor microenvironment in ways that could influence response to immunotherapies. A limitation of the study is that we tested a single TNBC model (MDA-MB-231). Future studies will extend the analysis to include additional TNBC models or patient-derived xenografts (PDXs). Expanding future studies to include multiple TNBC models or PDXs could provide a more comprehensive understanding of MB/OV treatment efficacy across diverse TNBC subtypes [63].

## 4. Materials and Methods

### 4.1. Cell Culture

MDA-MB-231 TNBC cells and A549 lung cancer cells were obtained from the American Type Culture Collection (ATCC, Rockville, MD, USA). MDA-MB-231 cells were labeled with firefly luciferase through lentiviral transduction and the MDA-MB-231-luc clones were selected for in vivo experiments based on photon flux (>10^9^) of bioluminescence image. MDA-MB-231-luc cells were grown in Dulbecco’s modified Eagle’s medium (Hyclone) supplemented with 10% FBS (Hyclone, Logan, UT, USA), and 100 units/mL penicillin supplemented with 1 mg/mL streptomycin (Hyclone). A549 cells were grown in DMEM:F12 (Hyclone) supplemented with 10% FBS (Hyclone), and 100 units/mL penicillin supplemented with 1 mg/mL streptomycin (Hyclone). All cells were grown at 37 °C, in a 5% CO_2_ in 95% atmosphere incubator.

### 4.2. Oncolytic Adenovirus Ad 5/3 Production and Titration

The Oncolytic Adenovirus Ad5/3-RGD D24 is a next-generation conditionally replicating Adenovirus that incorporates a 24-base pair deletion (D24) in the nucleotide sequence encoding the Rb-binding domain of the immediate early gene E1A, resulting in a virus deficient for viral replication in normal cells, and it is an Ad5/3 serotype chimera with an RGD modified fiber [64]. The Ad5/3-RGD D24 was amplified and purified for in vivo use by ViraQuest Inc., North Liberty, IA, USA and viral titers were determined by Tissue Culture Infectious Dose 50 (TCID50).

### 4.3. Incorporation of the OVs in the Imagent^®^ Microbubbles

Imagent^®^ MBs (Vesselon, Norwalk, CT, USA) are a perfluorohexane/nitrogen-filled agent with a phospholipid shell. These MBs are FDA-approved as a lyophilized product ready to be reconstituted with sterile water for injection. Each vial of reconstituted Imagent^®^ suspension contains an average of 1.37 × 10^10^ MBs. The microspheres have a mean volume-weighted median diameter of 6 μm. Imagent^®^ MBs were reconstituted in the presence of 2.8 × 10^10^ PFU. The viruses adsorbed on the MB surface were removed by treating with ten volumes of human serum complement at 60 mg/mL (Creative lab, Milpitas, CA, USA, CTS-006), and then by washing the MBs into sterile saline before injection. The final crema (300 µL) was counted using a hemocytometer and diluted with 2 mL of saline to a concentration of 5 × 10^7^ PFU/100 µL. One aliquot of the preparation was used for OV titration.

### 4.4. Mice, Human Tumor Xenografts, and Experimental Treatments

The animal studies were performed in accordance with the National Institutes of Health recommendations and following the approval of the Institutional Animal Care and Use Committee (IACUC) at the University of Mississippi Medical Center. Animal care and humane use and treatment of mice were in strict compliance with (i) institutional guidelines, (ii) the Guide for the Care and Use of Laboratory Animals (National Academy of Sciences, Washington, DC, USA, 1996), and (iii) the Association for Assessment and Accreditation of Laboratory Animal Care International (Rockville, MD, USA, 1997).

For our studies, we used Hu-CD34^+^ NSG-SGM3, which are NSG-SGM3 (NOD.Cg-*Prkdc^scid^ Il2rg^tm1Wjl^* Tg(CMV-IL3,CSF2,KITLG)1Eav/MloySzJ, Stock No. 013062) mice engrafted with human cord blood-derived CD34^+^ hematopoietic stem cells. NSG-SGM3 mice combine the features of the highly immunodeficient NOD acid gamma (NSG) mouse platform with transgenic expression of human IL3, GM-CSF (CSF2), and SCF (KITLG) cytokines for improved development of human myeloid lineages and regulatory T cell populations.

Forty-eight Hu-CD34^+^ NSG-SGM3 mice were injected with MDA-MB-231-luc human TNBC cells in their flanks (right and left, 5 × 10^6^ cells/mouse/flank in Saline: Matrigel Growth Factor Reduced 1:1). When tumors reached 150–200 mm^3^ by caliper measurement, mice were randomized by body weight and randomly assigned to one of the following 6 groups: (1) CTRL saline (IV); (2) OVs (IT); (3) OVs (IV); (4) Pembrolizumab (IP); (5) OVs/MBs (IV); (6) OVs/MBs (IV) + Pembrolizumab (IP) (Table 1). Mice were assigned to a treatment group using a random number generator. Once assigned to the groups, mice ears were punctured for identification.

Mice were anesthetized using a VetEquip (Pleasanton, CA, USA) IMPAC6 anesthesia machine. Mice were transferred in a BSL2 hood on a heating pad and anesthesia was continued using a nose cone to provide the isofluorane during the procedure. Mice were treated according to the randomization group of assignment as follows. Mice in Groups 1, 2, 3, 5 and 6 received three injections, one dose every 5 days (Q5Dx3) of either saline control (IV), OVs (IT), OVs (IV), or OV/MBs using an insulin syringe with a 29 G needle. The mice in groups 2 and 3 received 5 × 10^7^ PFU/mouse/50 µL of OVs by IT or IV. The mice in groups 5 and 6 received 5 × 10^7^ PFU/mouse/100 µL of MBs/OVs in the lateral tail vein followed by US on the tumor on the right flank for 7 min using a Mindray TE7 ultrasound machine (Mahwah, NJ, USA) and a 2.2 MHz probe set at 0.33 Mechanical Index (MI), with a transient pulse of 1.3 MI every 10 s. The mice in groups 1, 4, and 6 received six intraperitoneal (IP) injections, one dose every 5 days (Q5Dx6) of saline or Pembrolizumab (Selleckchem, anti-PD-1, A2005). The first injection was performed at 10 mg/kg in saline and the following injections at 5 mg/kg in saline.

### 4.5. Tumor Volume Measurements

Tumors were measured once a week using a digital caliper to measure the greatest longitudinal diameter (length) and the greatest transverse diameter (width). The volume was calculated by the modified ellipsoidal formula: V = ½ (Length × Width^2^). Tumor volumes were also calculated by IVIS imaging. Briefly, a fresh stock of Luciferin was prepared at 15 mg/mL in saline and sterile filtered through a 0.2 μm filter. Each mouse was weighed and received 150 mg/kg by IP (Luciferin sodium salt, Regis Technologies, Morton Groves, IL, USA). Mice were anesthetized and transferred into the IVIS imaging machine and images were acquired. After the procedure, mice were transferred to their cages and kept under observation until fully awake. Images were analyzed by measuring ROI Avg Radiance [p/s/cm^2^/sr].

### 4.6. Tumor and Organ Collection

At the experimental endpoint (4 weeks from the first treatment), mice were sacrificed and tumors and organs were collected. Spleens were processed to isolate splenocytes that were stored in liquid nitrogen for further analysis. Right flank and left flank tumors, lungs, heart, liver, and kidneys were collected and fixed in 10% neutral buffered formalin for 72 h and then stored in saline.

### 4.7. Hematoxylin and Eosin Staining and Immunohistochemical Analysis (IHC)

Tumors were dissected, processed, and embedded in paraffin blocks. Sections were cut at 4 µm in thickness and placed on electromagnetically + charged slides to prevent tissue detachment. Hematoxylin and eosin staining was performed for routine histopathological evaluation, which included evaluation of necrosis. Immunohistochemistry was performed using the avidin–biotin–peroxidase methodology, according to the manufacturer’s instructions (Vectastain ABC Elite Kit, Vector Laboratories, Burlingame, CA, USA). Our modified protocol includes deparaffination in xylenes, rehydration through descending grades of alcohol (100%, 85%, 70%), up to water, non-enzymatic antigen retrieval with 0.01 M sodium citrate buffer pH 6.0 at 95 °C for 30 min, endogenous peroxidase quenching with 3% H_2_O_2_ in methanol, and blocking with normal goat serum (for rabbit recombinant antibodies). Primary antibodies were incubated overnight at room temperature in a humidified chamber. For viral detection, a primary rabbit monoclonal anti-Adenovirus type 5 Hexon antibody was used (Clone EPR28237-57, 1:500 dilution, Abcam, Waltham, MA, USA). Primary antibodies for the detection of immune cells included a rabbit recombinant multiclonal anti-CD4 (RM1013, 1:1000 dilution, Abcam), a rabbit recombinant monoclonal anti-CD8a (Clone EPR21769, 1:2000 dilution, Abcam), and a rabbit monoclonal anti-CD25/IL-2Ra (Clone SP176, 1:100 dilution, Abcam). After rinsing in phosphate buffer saline solution (PBS), sections were incubated with biotinylated secondary antibodies for 1 h at room temperature, followed by incubation with avidin–biotin–peroxidase complexes for 1 h. Finally, the peroxidase was developed with diaminobenzidine (DAB Tablets, Boehringer, Mannheim, Germany) for 3 min, and the sections were counterstained with Hematoxylin and mounted with Permount (Fisher Scientific, Pittsburgh, PA, USA). Photomicrographs were taken with an Olympus DP72 Digital Camera using an Olympus BX70 microscope (Olympus, Center Valley, PA, USA).

Adenovirus-5 Hexon was evaluated as a cytoplasmic expression. CD4, CD8, and CD25 were evaluated as membranous expressions of tumor-infiltrating lymphocytes for immunolabeling frequency (based on the percentage of positively immunolabeled lymphocytes). Scores were assigned as 0 (0%), 1 (1–25%), 2 (26–50%), 3 (51–75%), 4 (76–100%).

### 4.8. Statistical Analysis

The number of Hu-CD34^+^ NSG-SGM3 required for our study was determined by performing a power calculation for two-way ANOVA, which showed that a sample size of 8 mice per group would detect a difference of 20% or more in the concentration of virus present in the organ target by the ultrasound and the control not targeted (IVIS imaging of luciferase) with a level of statistical significance level *p* ≤ 0.05 and power 95%. This difference has been observed in similar comparisons by experiments performed in our laboratory [3]. Statistical analysis was performed using GraphPad Prism 10 statistical software (Graphpad, Inc., La Jolla, CA, USA). One- and two-way analysis of variance (ANOVA) with Tukey or Bonferroni multiple comparisons post-test was used to determine the statistical significance of the differences between experimental groups. Multiple one-tailed *t*-tests were also used to compare the treatment groups. *P*-values of less than 0.05 were considered statistically significant.

## 5. Conclusions

The data gathered in our study by IVIS whole-animal body luminescence did not objectively reflect the beneficial effects of oncolytic viral treatment on the size of tumors implanted in the humanized mice. Notwithstanding the high luminescence (probably due to the most peripheral portion of the live tumor), we found larger necrosis, a lower ratio of CD4^+^/CD8^+^ TILs, and a high ratio of CD8^+^/CD25^+^ in the TNBC tumors that were concomitantly treated with MB/oncolytic viruses and pembrolizumab than control tumors, which is in agreement with previously published human studies of TNBC [38,40,41].

## Figures and Tables

**Table 1:** Experimental and control animal groups.

Groups	1	2	3	4	5	6
Agents	MBs	OVs	OVs	Pembrolizumab	OVs/MBs	OVs/MBs + Pembrolizumab
Route	IV	IT	IV	IP	IV	IV/IP
US (right flank)	YES	NO	NO	NO	YES	YES

**Table 2:** MB count in the vial of Imagent^®^ microbubbles used for the experiments.

Dates	Average MBs/mL	+/−SE	MBs (Total in 2.3 mL)	MBs/100 µL/Mouse
Dec.06.2022	4.85 × 10^8^	2.50 × 10^7^	1.12 × 10^9^	4.85 × 10^7^
Dec.14.2022	9.10 × 10^8^	5.00 × 10^7^	2.09 × 10^9^	9.10 × 10^7^
Dec.19.2022	3.56 × 10^9^	3.38 × 10^8^	8.19 × 10^9^	3.56 × 10^8^

**Table 3:** Titration of OVs recovered from the MB preparation.

Dates	Average PFU/mL	+/−SE	PFUs (Total in 2.3 mL)	PFU/100 µL/Mouse
Dec.06.2022	9.80 × 10^5^	1.10 × 10^5^	2.25 × 10^6^	9.80 × 10^4^
Dec.14.2022	9.80 × 10^5^	1.10 × 10^5^	2.25 × 10^6^	9.80 × 10^4^
Dec.19.2022	1.09 × 10^9^	1.10 × 10^8^	2.51 × 10^9^	1.09 × 10^8^

**Table 4:** Average tumor size/necrosis ratio of right and left flank xenografts. (SE) standard error.

Groups	Injection Route	Average Tumor Size/Tumor Necrosis Ratio (Right Flank)	SE	Average Tumor Size/Tumor Necrosis Ratio (Left Flank)	SE
1. MBs	IV	4.30	1.05	4.09	1.11
2. OVs	IT	5.72	0.85	4.85	0.94
3. OVs	IV	2.91	0.11	4.94	0.89
4. Pembro	IP	4.52	1.13	5.20	1.32
5. MBs/OVs	IV	2.32	0.30	2.49	0.34
6. MBs/OVs + Pembro	IV	1.78	0.27	2.91	0.17

**Table 5:** *p*-values of *t*-test comparisons of tumor size/necrosis ratios on right side treated tumors. Significance level α < 0.05; * < 0.05; (ns) not significant.

Treated Tumors Right Flank	OV (IT)	OV (IV)	Pembro (IP)	MB/OV/US (IV)	MB/OV/US (IV) + Pembro (IP)
MB (IV)	0.155 (ns)	0.131 (ns)	0.445 (ns)	0.05	0.031 (* *p* < 0.05)
OV (IT)		0.010 (* *p* < 0.05)	0.213 (ns)	0.002 (* *p* < 0.05)	0.001 (* *p* < 0.05)
OV (IV)			0.147 (ns)	0.063 (ns)	0.002 (* *p* < 0.05)
Pembro (IP)				0.064 (ns)	0.045 (* *p* < 0.05)
MB/OV/US (IV)					0.113 (ns)

**Table 6:** *p*-values of *t*-test comparisons of tumor size/necrosis ratios on left side untreated tumors. Significance level α < 0.05; * < 0.05; (ns) not significant.

Untreated Tumors Left Flank	OV (IT)	OV (IV)	Pembro (IP)	MB/OV (IV)	MB/OV (IV) + Pembro (IP)
MB (IV)	0.303 (ns)	0.287 (ns)	0.276 (ns)	0.100 (ns)	0.183 (ns)
OV (IT)		0.474 (ns)	0.420 (ns)	0.024 (* *p* < 0.05)	0.05
OV (IV)			0.445 (ns)	0.011 (* *p* < 0.05)	0.027 (* *p* < 0.05)
Pembro (IP)				0.055 (ns)	0.103 (ns)
MB/OV/US (IV)					0.172 (ns)

**Table 7:** *p*-values of multivariate ANOVA comparison analysis with Tukey multiple comparisons post-test on the expression of CD4^+^ T cells in treated tumors (right side). Significance level α < 0.05; * < 0.05; ** < 0.01; (ns) not significant. In brackets are reported the 95% confidence intervals (CI_95_) of the comparisons.

Treated Tumors Right Flank	OV (IT)	OV (IV)	Pembro (IP)	MB/OV/US (IV)	MB/OV/US (IV) + Pembro (IP)
MB (IV)	** < 0.01 (2.76–1.21)	* < 0.05 (−1.65–0.02)	0.899 (ns)	<0.01 (2.55–0.94)	** < 0.01 (3.25–1.57)
OV (IT)		** < 0.01 (0.35–1.98)	** < 0.01 (1.03–2.47)	0.899 (ns)	0.551 (ns)
OV (IV)			0.202 (ns)	* < 0.05 (0.09–1.77)	** < 0.01 (0.72–2.47)
Pembro (IP)				** < 0.01 (0.77–2.27)	** < 0.01 (1.39–2.98)
MB/OV/US (IV)					0.149 (ns)

**Table 8:** *p*-values of multivariate ANOVA comparison analysis with Tukey multiple comparisons post-test on the expression of CD8^+^ T cells in treated tumors (right side). Significance level α < 0.05; * < 0.05; ** < 0.01; (ns) not significant. In brackets are indicated the 95% confidence intervals (CI_95_) of the comparisons.

Treated Tumors Right Flank	OV (IT)	OV (IV)	Pembro (IP)	MB/OV/US (IV)	MB/OV/US (IV) + Pembro (IP)
MB (IV)	** < 0.01 (1.63–0.05)	0.536 (ns)	0.899 (ns)	** < 0.01 (3.68–1.64)	** < 0.01 (4.83–2.69)
OV (IT)		0.093 (ns)	** < 0.01 (0.84–2.72)	** < 0.01 (0.23–2.19)	** < 0.01 (1.28–3.34)
OV (IV)			0.110 (ns)	** < 0.01 (1.03–3.16)	** < 0.01 (2.08–4.31)
Pembro (IP)				** < 0.01 (2.01–3.98)	** < 0.01 (3.06–5.13)
MB/OV/US (IV)					* < 0.05

**Table 9:** *p*-values of multivariate ANOVA comparison analysis with Tukey multiple comparisons post-test on the expression of CD8^+^ T cells in untreated tumors (left side). Significance level α < 0.05; * < 0.05; ** < 0.01; (ns) not significant. In brackets are indicated the 95% confidence intervals (CI_95_) of the comparisons.

Untreated Tumors Left Flank	OV (IT)	OV (IV)	Pembro (IP)	MB/OV/US (IV)	MB/OV/US (IV) + Pembro (IP)
MB (IV)	0.899 (ns)	0.391 (ns)	0.899 (ns)	** < 0.01 (1.77–0.22)	** < 0.01 (2.11–0.48)
OV (IT)		0.678 (ns)	0.899 (ns)	* < 0.05 (0.10–1.60)	** < 0.01 (0.37–1.94)
OV (IV)			0.615 (ns)	0.391 (ns)	0.054 (ns)
Pembro (IP)				** < 0.01 (0.14–1.60)	** < 0.01 (0.40–1.94)
MB/OV/US (IV)					0.827 (ns)

**Table 10:** Average CD4^+^/CD8^+^ ratio and CCD8^+^/CD25^+^ ratio of intratumoral T-lymphocyte infiltrates in treated (right flank) and untreated tumors (left flank).

Groups	Route	CD4^+^/CD8^+^ Ratio Right Flank Tumor	SD	CD4^+^/CD8^+^ Ratio Left Flank Tumor	SD	CD8^+^/CD25^+^ Ratio Right Flank Tumor	SD	CD8^+^/CD25^+^ Ratio Left Flank Tumor	SD
1. MBs	IV	0	-	0	-	0	-	0	-
2. OVs	IT	1.16	0.33	0.75	0.50	0.56	0.13	0.39	0.19
3. OVs	IV	1.00	0.00	0.80	0.27	1.87	1.54	1.66	0.57
4. Pembro	IP	1.12	0.83	0.62	0.50	0.47	0.13	0.56	0.32
5. MBs/OVs	IV	0.69	0.18	0.66	0.25	3.5	0.70	4.00	0.00
6. MBs/OVs + Pembro	IV	0.66	0.08	0.60	0.22	4.66	0.57	3.50	1.0

**Table 11:** *p*-values of multivariate ANOVA comparison analysis with Tukey multiple comparisons post-test on the CD8^+^/CD25^+^ T-cell ratios of treated tumors (right flank). Significance level α < 0.05; * < 0.05; ** < 0.01; (ns) not significant. In brackets are indicated the 95% confidence intervals (CI_95_) of the comparisons.

Treated Tumors Right Flank	OV (IT)	OV (IV)	Pembro (IP)	MB/OV/US (IV)	MB/OV/US (IV) + Pembro (IP)
MB (IV)	0.899 (ns)	* < 0.05 (2.66–0.08)	0.899 (ns)	** < 0.01 (4.63–1.36)	** < 0.01 (5.58–2.74)
OV (IT)		* < 0.05 (2.56–0.04)	0.785 (ns)	** < 0.01 (1.32–4.53)	** < 0.01 (2.71–5.48)
OV (IV)			** < 0.01 (0.47–2.93)	0.054 (ns)	** < 0.01 (1.26–4.32)
Pembro (IP)				** < 0.01 (1.74–4.91)	** < 0.01 (3.13–5.85)
MB/OV/US (IV)					0.331 (ns)

## Data Availability

Data are available from the corresponding author upon reasonable request.
